# To Tap or to Explore?

**Published:** 1897-07-31

**Authors:** 


### TO TAP OR TO EXPLORE?

After tapping a case of ascites, how often the surgeon has to regret that he has learned but little by the proceeding, however much relief the patient may, for a time at least, have obtained. Why, then, should we perform paracentesis when, by making an opening about five times as long, the finger could be introduced, and the exact condition of affairs within the abdomen could be ascertained? Dr. Wallace, of Liverpool, goes further, and advocates a two-inch incision, through which a long, specially-made Ferguson's speculum can be introduced, by which means a view can be obtained of almost any part of the peritoneal cavity. The fact is, as he states, that paracentesis is a blind proceeding which is done in the dark, while an incision one and a half to two inches in length can be performed on surgical principles, and is, he considers, the less dangerous operation of the two. On the other hand, not only does incision give an opportunity for exploring the abdominal cavity by the finger or, as Dr. Wallace suggests, by the speculum, but it also is of far greater curative efficacy than mere paracentesis. The latter may be said never to cure. The physical conditions are altered for a moment, only the fluid being so rapidly re-formed. Incision followed by drainage is a very different affair, and if the origin of the effusion has been some inflammatory change in the peritoneum an actual cure may result. An incision "not only taps and drains the ascitic cavity, but also the vascular system of the whole peritoneal sac, so that for days the discharge is sero-sanguineous." It seems quite possible that even in some cases of hepatic enlargement due to alcoholism the effusion may be due to inflammatory changes. Cases are mentioned in which incision has given permanent relief. Dr. Wallace specially insists on the fact that for every case of ascites there is some immediate cause, generally in the peritoneal cavity, even when some more generally recognised cause, such as heart disease, may predispose to its occurrence, and that this local cause may sometimes be relieved by incision and drainage, whereas tapping leaves it just as it was before. It is a matter worth careful consideration.

